# Analysis of perturbed Boussinesq equation via novel integrating schemes

**DOI:** 10.1371/journal.pone.0302784

**Published:** 2024-05-17

**Authors:** Miguel Vivas-Cortez, Saima Arshed, Zahida Perveen, Maasoomah Sadaf, Ghazala Akram, Kashif Rehan, Komal Saeed

**Affiliations:** 1 Escuela de Ciencias Físicas y Matemáticas, Facultad de Ciencias Exactas y Naturales, Pontificia Universidad Católica del Ecuador, Apartado, Quito, Ecuador; 2 Department of Mathematics, University of the Punjab, Quaid-e-Azam Campus, Lahore, Pakistan; 3 Department of Mathematics, Lahore Garrison University, Lahore, Pakistan; 4 Department of Mathematics, University of Engineering and Technology, KSK Campus, Lahore, Pakistan; Federal University of Technology - Parana, BRAZIL

## Abstract

To analyze and study the behaviour of the shallow water waves, the perturbed Boussinesq equation has acquired fundamental importance. The principal objective of this paper is to manifest the exact traveling wave solution of the perturbed Boussinesq equation by two well known techniques named as, two variables $\left(\frac{G^{\prime }}{G},\frac{1}{G}\right)$ expansion method and generalized projective Riccati equations method. A diverse array of soliton solutions, encompassing periodic, bright solitons, singular solitons and bright singular solitons are obtained by the applications of proposed techniques. The constraint conditions for newly constructed solutions are also specified. To enhance comprehension, the numerical illustrations of constructed solutions have been represented using surface plots, 2D plots and density plots. The results delineated in this paper transcend existing analysis, offering a novel, well-structured, and modern perspective. The solutions obtained not only enrich understanding of shallow water wave models but also exhibit efficacy in providing detailed descriptions of their dynamics.

## 1 Introduction

The world around us contains nonlinear phenomena and to describe these phenomena, nonlinear partial differential equations play a vital role. It is not possible to deny the importance of nonlinear partial differential equations (NLPDEs). NLPDEs have been widely used to explain and investigate the physical phenomena occurring in the world. Their applications have provided fruitful results in all fields of natural and social sciences including engineering and biological sciences [1].

In recent years, researchers have paid much attention to finding the traveling wave solutions of NLPDEs. Solitary waves and solitons are the type of traveling waves that were first discovered by J. Scott Russell in 1834. Solitons have some unique properties e.g. solitons come into existence when nonlinear and dispersive effects are canceled in a medium. Soliton acts like a single wave with one crust. It retains its shape while traveling at a constant speed. Many researchers have worked hard for finding soliton solutions of many nonlinear evolution equations. The NLEEs are used to describe complex problems in various fields such as, plasma physics, mechanics of water waves, nonlinear optical fibers, control theory, describing oceanic and atmospheric influences, fluid mechanics, mathematical chemistry, biology, signal processing and many more [1, 2]. The methods of finding exact solutions of NLEEs using traveling wave transformations are a popular topic of research. Many useful methods have been employed for extracting solitary wave solutions, such as; improved $\text{tan}(\frac{\phi (\xi )}{2})$ expansion method [3], first integral method [4], modified auxiliary equation method [5], generalized exponential rational function (GERF) method [6], an inverse-$\frac{G^{\prime }}{G}$ expansion method [7], the Lie classical method and unified method [8], the modified generalized Riccati equation mapping approach [9], the modified generalized exponential rational function method and the extended function method [10], new modified generalized Riccati equation mapping approach [11] and others.

The fundamental aim of this research article is to investigate perturbed Boussinesq equation (PBE) as it holds significant importance in fluid dynamics, particularly in the context of water waves. This model yields soliton solutions, shock waves, and singular solutions. These solutions are very useful in studying different wave behavior under diverse conditions. The perturbed quantities occurring in the model allow the reader to study more complex wave behaviors, including solitons and other solutions. The perturbed Boussinesq equation, which is used to describe the propagation of waves in shallow water is being investigated in this article. This equation incorporates various effects such as refraction, diffraction, shoaling, and weak nonlinearity in fluid dynamics. It is integrable equation and possesses soliton solutions.

Boussinesq equation in various formats has been analyzed by many scholars through different techniques such, as the extended hyperbolic function method [12], modified auxiliary equation techniques [13], $\left(\frac{G^{\prime }}{G}\right)$ method [2] and approximate symmetry method [14].

The two variables $\left(\frac{G^{\prime }}{G},\frac{1}{G}\right)$ expansion method and generalized projective Riccati equations method are reliable and efficient approaches for obtaining new and novel solutions in the form of hyperbolic, trigonometric and rational functions. The extracted hyperbolic function solutions can further lead to bright soliton, dark soliton, Kink and anti-Kink solutions, singular solitons. The trigonometric solutions can be referred to as periodic solutions. All analytical methods have some limitations. The analytical techniques that are applied in this article also have some limitations. The proposed approaches extracted dark solitons, periodic waves and bright solitons. The approaches are strong and play an efficient role in finding solutions to variety of NLPDEs. The proposed techniques are widely employed in nonlinear dynamics and soliton theory, to create solitonic shapes. These techniques provide the full spectrum of soliton solutions.

The analytical methods that have been used in this article have significance importance in solving nonlinear partial differential equations. These methods have been used by many scholars to solve nonlinear equations, such as Konno-Oono equation [15], biological population model and KdV-Zakharov-Kznestsov equation [16] solved by two variables $\left(\frac{G^{\prime }}{G},\frac{1}{G}\right)$ expansion method. Whereas, generalized projective Riccati equations method is used to solve Klien-Fock-Gordon equation [17] and Lakshmanan Porsezian Daniel model [18].

This paper is divided into six sections: governing model is described in Section 2, Section 3 is about detailed explanation of above mentioned methods, Section 4 gives mathematical analysis of perturbed Boussinesq equation, Section 5 provides graphical illustrations and Section 6 concludes the whole paper.

## 2 Governing model (perturbed Boussinesq equation (PBE)) description

Boussinesq equation is the fundamental equation that is modeled for stability of waves by interaction of surface waves over shallow water waves. Boussinesq describes that this wave maintains its shape by balancing between precipitous effect of nonlinearity and smooth effect of dispersion.

The Boussinesq equation is given as

$$\begin{array}{c} S_{tt}-S_{xx}-\alpha S_{xx}^{2}+\omega S_{xxxx}=0, \end{array}$$ (1)

where *S*(*x*, *t*) is a function of *x* and *t* representing surface elevation, *α* and *ω* are treated as constant parameters.

When *ω* > 0, then we end up with, a linearly stable, and the numerical computable equation Eq (1) known as Good Boussinesq (GB) equation. The solitary waves described by the GB equation solely occur for a finite range of velocities and can merge into one solitary wave [19].

If the sign associated to *ωS*_*xxxx*_ is changed i.e *ω* < 0, Eq (1) becomes the well-known bad Boussinesq equation. It is used to describe a two-dimensional flow of shallow water over a flat bottom, assuming that the water waves have small amplitudes. Bad Boussinesq-type has its importance from both mathematical and physical points of view, but the research results on its initial boundary value problems are scarce. A part of the reason for the paucity of the results is due to the properties of the linear part of Eq (1) that are so bad that the traditional mathematical methods cease to be effective [20].

With the advancement in the Boussinesq equation, many new forms of Boussinesq equations are developed and are used frequently by researchers. In this respect, the perturbed Boussinesq equation (PBE) comes out, as

$$\begin{array}{c} S_{tt}-\kappa^{2}S_{xx}+jS_{xx}^{2n}+hS_{xxxx}=\omega S_{xx}+\rho S_{xxxx}, \end{array}$$ (2)

where, *ρ* indicates stabilization term and *ω* represents coefficient of dissipation [21]. PBE is a popular nonlinear evolution equation and it has been widely implemented in coastal, harbor, water and oceanic engineering. It helps in forecasting waves in coastal areas, breaking of waves, interaction of waves, shoreline circulation in intense and normal weather conditions [2]. Moreover it is used to model tsunami waves, oscillations of tidal waves and also to model the characteristics of shallow water waves which occur at beaches, lakes and in rivers.

PBE model has attracted the attention of many researchers. In [22] the perturbed Boussinesq equation is investigated using generalized Kudryashov method and sine-Gordon expansion method. A study is also conducted on new perturbed conformable Boussinesq-like equations to deduce soliton solutions [23]. In [13], new approximate symmetry method is applied on (2+1)-dimensional perturbed Boussinesq equation and new soliton solutions have been derived.

## 3 Description of methods

This section contains the detailed description of two proposed analytical methods.

### 3.1 Method 1: Two variables $\left(\frac{G^{\prime }}{G},\frac{1}{G}\right)$ expansion method

To have a full grasp of two variables $\left(\frac{G^{\prime }}{G},\frac{1}{G}\right)$ expansion method following preliminary points are important to keep in mind:

***Point 1.*** The linear ordinary differential equation of 2^*nd*^ order is considered, as

$$\begin{array}{c} G^{\prime \prime }\left(\eta \right)+\lambda G\left(\eta \right)=\mu . \end{array}$$ (3)

Fix $E=\frac{G^{\prime }}{G}$ and $F=\frac{1}{G}$. Moreover, *E* and *F* obey the following ODEs

$$\begin{array}{c} E^{\prime }=-E^{2}+\mu F-\lambda ,\;F^{\prime }=-EF, \end{array}$$ (4)

where λ and *μ* are treated as constants.

***Point 2.*** When λ < 0, the general solution of Eq (3) is obtained, as

$$\begin{array}{c} G\left(\eta \right)=B_{1}\;\text{sinh}\left(\eta \sqrt{-\lambda }\right)+B_{2}\;\text{cosh}\left(\eta \sqrt{-\lambda }\right)+\frac{\mu }{\lambda }, \end{array}$$ (5)

where *B*_1_ and *B*_2_ indicate arbitrary constants. Consequently, *F* is taken as

$$\begin{array}{c} F^{2}=\frac{-\lambda }{\lambda^{2}\sigma_{1}+\mu^{2}}\left(E^{2}-2\mu F+\lambda \right), \end{array}$$ (6)

where $\sigma_{1}=B_{1}^{2}-B_{2}^{2}$.

***Point 3***. When λ > 0, then the general solution of Eq (3) is obtained, as

$$\begin{array}{c} G\left(\eta \right)=B_{1}\text{sin}\left(\eta \sqrt{\lambda }\right)+B_{2}\;\text{cos}\left(\eta \sqrt{\lambda }\right)+\frac{\mu }{\lambda }, \end{array}$$ (7)

where *F* is considered as

$$\begin{array}{c} F^{2}=\frac{\lambda }{\lambda^{2}\sigma_{2}-\mu^{2}}\left(E^{2}-2\mu F+\lambda \right), \end{array}$$ (8)

where $\sigma_{2}=B_{1}^{2}+B_{2}^{2}$.

***Point 4***. When λ = 0, then solution of Eq (3) is obtained, as

$$\begin{array}{c} G\left(\eta \right)=\frac{\mu }{2}\eta^{2}+B_{1}\eta +B_{2}, \end{array}$$ (9)

the value of *F* has the form

$$\begin{array}{c} F^{2}=\frac{1}{B_{1}^{2}-2\mu B_{2}}\left(E^{2}-2\mu F\right). \end{array}$$ (10)

The NLEE is considered, as

$$\begin{array}{c} R(S,S_{t},S_{x},S_{xx},...)=0, \end{array}$$ (11)

where *R* represents a polynomial in *S*(*x*, *t*) and its partial derivatives. Following are the important steps of two variable $\left(\frac{G^{\prime }}{G},\frac{1}{G}\right)$ expansion method.

***Fist step.*** The following traveling wave transformation is used

$$\begin{array}{c} S(x,t)=p(\eta ),\;\;\eta =x-vt, \end{array}$$ (12)

where *v* represents a constant velocity. After applying this transformation, Eq (11) is converted to an ODE as

$$\begin{array}{c} Q(p^{\prime },p^{\prime \prime },p^{\prime \prime \prime }\ldots )=0, \end{array}$$ (13)

where *Q* shows a polynomial of *p*(*η*) and its all derivatives with respect to *η*.

***Second step.*** It is assumed that the Eq (13) has solution of the following form

$$\begin{array}{c} p\left(\eta \right)=\sum_{r=0}^{M}c_{r}E^{r}+\sum_{r=1}^{M}d_{r}E^{r-1}F, \end{array}$$ (14)

where *c*_*r*_(*r* = 0, 1, 2, …, *M*) and *d*_*r*_(*r* = 1, 2, …, *M*) are constants, which are determined later.

***Third step.*** In order to determine the integer *M* in Eq (14), the homogenous balancing is employed, through that the highest-order derivative and the nonlinear term occurring in the equation are compared.

***Fourth step.*** By plugging Eq (14) into Eq (13) along with Eqs (4) and (6), the left-hand side of Eq (13) is transferred into a polynomial in *E* and *F*, in which the degree of *F* is not greater than 1. Setting each coefficients of gained polynomial to zero. The homogenous system of algebraic equations is obtained. Upon solving the system, the values of *c*_*r*_, *d*_*r*_, *v*, *μ*, *B*_1_, *B*_2_ and λ for the case of λ < 0 are determined.

***Fifth step.*** Following the same procedure as explained in *Fourth step.*, inserting Eq (14) into Eq (13) besides with Eqs (4) and (8) for the case λ > 0 and for the case λ = 0 inserting Eq (14) into Eq (13) with Eqs (4) and (10)), the values of arbitrary constants *c*_*r*_, *d*_*r*_, *v*, *μ*, *B*_1_, *B*_2_ and λ are extracted. After substituting the values of arbitrary constants in Eq (14), the exact solutions of Eq (13) have been obtained which are expressed by trigonometric functions, hyperbolic functions or rational functions for λ > 0, λ < 0 or λ = 0, respectively.

### 3.2 Method 2: Generalized projective Riccati equations method

***First step.*** According to Method 2, the general solution of Eq (13) has the form

$$\begin{array}{c} p\left(\eta \right)=a_{0}+\sum_{r=1}^{M}A^{r-1}\left(\eta \right)[a_{r}A\left(\eta \right)+b_{r}B\left(\eta \right)], \end{array}$$ (15)

where *a*_0_, *a*_*r*_ and *b*_*r*_ are constants which are determined later. The functions *A*(*η*) and *B*(*η*) satisfy the following ordinary differential equations

$$A^{\prime }\left(\eta \right)=\epsilon A\left(\eta \right)B\left(\eta \right),$$

$$\begin{array}{c} B^{\prime }\left(\eta \right)=H+\epsilon B^{2}\left(\eta \right)-\delta A\left(\eta \right), \end{array}$$ (16)

where

$$\begin{array}{c} B^{2}\left(\eta \right)=-\epsilon (H-2\delta A\left(\eta \right)+\frac{\delta^{2}+i}{H}A^{2}\left(\eta \right)),\;\epsilon =\pm 1, \end{array}$$ (17)

valid for values of *i* = ±1 where *H* and *δ* are nonzero constants.

When *δ* = 0 and *H* = 0 then solution of Eq (15) has the following form

$$\begin{array}{c} p\left(\eta \right)=\sum_{r=0}^{M}a_{r}B^{r}\left(\eta \right), \end{array}$$ (18)

*B*(*η*) satisfies the ordinary differential equation

$$\begin{array}{c} B^{\prime }\left(\eta \right)=B^{2}\left(\eta \right). \end{array}$$ (19)

***Second step.*** In order to determine the integer *M* in Eq (15), the homogenous balancing is employed, through that the highest-order derivative and the nonlinear term of the equation are compared.

***Third step.*** When *H* ≠ 0, Eq (15) is inserted along with Eqs (16) and (17) into Eq (13) and for the case when *H* = 0 and *δ* = 0, Eq (18) is inserted along with Eq (19) into Eq (13). A system of algebraic equations is obtained by setting every coefficient of *A*^*r*^(*η*)*B*^*q*^(*η*)(*r* = 0, 1, …, *q* = 0, 1) to zero. The system can be solved to obtain the values of *a*_0_, *a*_*r*_, *b*_*r*_, *v*, *δ* and *Y*.

***Fourth step.*** Different cases for the solution of Eq (16) are given, as follows [19]:

**Case 1:** For *ϵ* = −1, *i* = −1, *H* > 0,

$$\begin{array}{c} A_{1}\left(\eta \right)=\frac{H\text{sech}(\sqrt{H}\eta )}{\delta \text{sech}(H\eta )+1},\;B_{1}\left(\eta \right)=\frac{\sqrt{H}\;\text{tanh}\left(H\eta \right)}{\delta \text{sech}(\sqrt{H}\eta )+1}. \end{array}$$ (20)

For *ϵ* = −1, *i* = 1, *H* > 0,

$$\begin{array}{c} A_{2}\left(\eta \right)=\frac{H\text{csch}(\sqrt{H}\eta )}{\delta \text{csch}(\sqrt{H}\eta )+1},\;B_{2}\left(\eta \right)=\frac{\sqrt{H}\text{coth}\left(\sqrt{H}\eta \right)}{\delta \;\text{csch}(\sqrt{H}\eta )+1}. \end{array}$$ (21)

**Case 2:** For *ϵ* = 1, *i* = −1, *H* > 0,

$$\begin{array}{c} A_{3}\left(\eta \right)=\frac{H\;\text{sec}\;(\sqrt{Y}\eta )}{\delta \;\text{sec}\;(\sqrt{H}\eta )+1},\;B_{3}\left(\eta \right)=\frac{\sqrt{H}\;\;\text{tan}\;\left(\sqrt{H}\eta \right)}{\delta \;\text{sec}\;(\sqrt{H}\eta )+1}. \end{array}$$ (22)

For *ϵ* = 1, *i* = 1, *H* > 0,

$$\begin{array}{c} A_{4}\left(\eta \right)=\frac{H\;\text{csc}\;(\sqrt{H}\eta )}{\delta \;\text{csc}\;(\sqrt{H}\eta )+1},\;B_{4}\left(\eta \right)=-\frac{\sqrt{H}\;\text{cot}\;\left(\sqrt{H}\eta \right)}{\delta \;\text{csc}\;(\sqrt{H}\eta )+1}. \end{array}$$ (23)

**Case 3:** For *H* = *δ* = 0,

$$\begin{array}{c} A_{5}\left(\eta \right)=\frac{G}{\eta },\;B_{5}\left(\eta \right)=\frac{1}{\epsilon \eta }, \end{array}$$ (24)

where *G* ≠ 0.

***Fifth step.*** Lastly, exact solutions of Eq (11) are obtained by plugging the values *a*_0_, *a*_*j*_, *b*_*j*_, where *j* = (1, 2, 3…, *M*) *Y*, *δ* and *v* along with Eqs (20)–(24) into Eq (15).

## 4 Extraction of solutions for the proposed model

This section provides the exact solutions of perturbed Boussinesq equation by applying the two variable expansion $\left(\frac{G^{\prime }}{G},\frac{1}{G}\right)$-method and the generalized projective Riccati equations method. The obtained solutions may be effective in providing detail description of shallow water waves models. In order to apply these methods, the following traveling wave transformation is considered.

$$\begin{array}{c} S(x,t)=p(\eta )\;\text{where}\;\eta =x-vt. \end{array}$$ (25)

In this paper, Eq (2) is solved for *n* = 1. The perturbed Boussinesq equation is converted into following ordinary differential equation by employing the wave transformation (25), as

$$\begin{array}{c} v^{2}p^{\prime \prime }-\kappa^{2}p^{\prime \prime }+j{\left(p^{2}\right)}^{\prime \prime }+hp^{\prime \prime \prime \prime }=\omega p^{\prime \prime }+\rho p^{\prime \prime \prime \prime }. \end{array}$$ (26)

Upon integrating Eq (26) twice and taking the constants of integration to be zero, Eq (26) takes the form

$$\begin{array}{c} \left(v^{2}-\kappa^{2}-\omega \right)p+jp^{2}+\left(h-\rho \right)p^{\prime \prime }=0. \end{array}$$ (27)

Application of both methods on Eq (27) is discussed in the following subsections.

### 4.1 Application of method 1 for PBE

This subsection is devoted for the application of two variable expansion $\left(\frac{G^{\prime }}{G},\frac{1}{G}\right)$-method on PBE. Homogenous balancing principle gives *M* = 2. Substituting *M* = 2 in Eq (14), the following form of solution is obtained.

$$\begin{array}{c} p\left(\eta \right)=c_{0}+c_{1}E+c_{2}E^{2}+d_{1}F+d_{2}EF. \end{array}$$ (28)

Putting Eq (28) into Eq (27) and utilizing Method 1, the following solution sets for two cases of λ have been derived.

#### 4.1.1 Case 1: λ < 0

Hyperbolic solutions will be obtained in this case. The following solution sets are extracted by applying Method 1.

1^*st*^ **solution set**

$$\begin{array}{ccc} & & c_{0}=-\frac{2\lambda (h-\rho )}{j},\;c_{1}=0,\;d_{1}=\frac{3\mu (h-\rho )}{j},\;d_{2}=-\frac{3\left(h-\rho \right)\sqrt{\lambda^{2}\sigma_{1}+\mu^{2}}}{\sqrt{-\lambda }j},\\ & & c_{2}=-\frac{3(h-\rho )}{j}. \end{array}$$

2^*nd*^ **solution set**

$$\begin{array}{ccc} & & c_{0}=-\frac{2\lambda (h-\rho )}{j},\;c_{1}=0,\;d_{1}=0,\;d_{2}=0,\;c_{2}=-\frac{6(h-\rho )}{j},\;\mu =0. \end{array}$$

3^*rd*^ **solution set**

$$\begin{array}{ccc} & & c_{0}=-\frac{2\lambda (h-\rho )}{j},\;c_{1}=0,\;d_{1}=0,\;d_{2}=-\frac{3\sqrt{-\lambda \sigma_{1}}\left(h-\rho \right)}{j},\;c_{2}=-\frac{3(h-\rho )}{j},\;\mu =0. \end{array}$$

The hyperbolic solutions extracted by 1^*st*^ **solution set** are obtained, as

$$\begin{array}{c} \begin{array}{cc} S_{1}\left(x,t\right)= & \frac{3\mu (h-\rho )}{j(B_{1}\;\text{sinh}\;(\sqrt{-\lambda }\eta )+B_{2}\;\text{cosh}\;(\sqrt{-\lambda }\eta )+\frac{\mu }{\lambda })}-\frac{2\lambda (h-\rho )}{j}\\ & -\frac{3\left(h-\rho \right)(B_{2}\sqrt{-\lambda }\;\;\text{sinh}\;(\sqrt{-\lambda }\eta )+B_{1}\sqrt{-\lambda }\;\;\text{cosh}\;(\sqrt{-\lambda }\eta ){)}^{2}}{j(B_{1}\;\text{sinh}\;(\sqrt{-\lambda }\eta )+B_{2}\;\text{cosh}\;(\sqrt{-\lambda }\eta )+\frac{\mu }{\lambda }{)}^{2}}\\ & -\frac{3\left(h-\rho \right)\sqrt{(B_{1}^{2}-B_{2}^{2})\lambda^{2}+\mu^{2}}(B_{2}\sqrt{-\lambda }\;\text{sinh}\;(\sqrt{-\lambda }\eta )+B_{1}\sqrt{-\lambda }\;\text{cosh}\;(\sqrt{-\lambda }\eta ))}{j\sqrt{-\lambda }(B_{1}\;\text{sinh}\;(\sqrt{-\lambda }\eta )+B_{2}\;\text{cosh}\;(\sqrt{-\lambda }\eta )+\frac{\mu }{\lambda }{)}^{2}}. \end{array} \end{array}$$ (29)

Particularly, by taking *B*_1_ = 0 and *B*_2_ = 1, Eq (29) yields the following solutions, as

$$\begin{array}{ccc} S_{1}\left(x,t\right) & = & -\frac{2\lambda (h-\rho )}{j}+\frac{3\mu (h-\rho )}{j(\frac{\mu }{\lambda }+\;\text{cosh}\;(\sqrt{-\lambda }\eta ))}+\frac{3\lambda \left(h-\rho \right)\;{\text{sinh}}^{2}\;(\sqrt{-\lambda }\eta )}{j{\left(\frac{\mu }{\lambda }+\;\text{cosh}\;(\sqrt{-\lambda }\eta )\right)}^{2}}\\ & & -\frac{3\left(h-\rho \right)\sqrt{\mu^{2}-\lambda^{2}}\;\text{sinh}\;(\sqrt{-\lambda }\eta )}{j{\left(\frac{\mu }{\lambda }+\;\text{cosh}\;(\sqrt{-\lambda }\eta )\right)}^{2}}. \end{array}$$ (30)

Again by setting *B*_1_ = 1 and *B*_2_ = 0, Eq (29) gives the solutions, as

$$\begin{array}{ccc} S_{1}\left(x,t\right) & = & -\frac{2\lambda (h-\rho )}{j}-\frac{3\left(h-\rho \right)\sqrt{\lambda^{2}+\mu^{2}}\;\text{cosh}(\sqrt{-\lambda }\eta )}{j{\left(\frac{\mu }{\lambda }+\text{sinh}(\sqrt{-\lambda }\eta )\right)}^{2}}+\frac{3\lambda \left(h-\rho \right){\text{cosh}}^{2}(\sqrt{-\lambda }\eta )}{j{\left(\frac{\mu }{\lambda }+\text{sinh}(\sqrt{-\lambda }\eta )\right)}^{2}}\\ & & +\frac{3\mu (h-\rho )}{j(\frac{\mu }{\lambda }+\text{sinh}(\sqrt{-\lambda }\eta ))}. \end{array}$$ (31)

The hyperbolic solutions extracted by 2^*nd*^ **solution set** are obtained, as

$$\begin{array}{c} S_{2}\left(x,t\right)=-\frac{2\lambda (h-\rho )}{j}-\frac{6\left(h-\rho \right)(B_{2}\sqrt{-\lambda }\text{sinh}(\sqrt{-\lambda }\eta )+B_{1}\sqrt{-\lambda }\text{cosh}(\sqrt{-\lambda }\eta ){)}^{2}}{j(B_{1}\text{sinh}(\sqrt{-\lambda }\eta )+B_{2}\text{cosh}(\sqrt{-\lambda }\eta )+\frac{\mu }{\lambda }{)}^{2}}. \end{array}$$ (32)

Taking *B*_1_ = 0 and *B*_2_ = 1, Eq (32) produces the following hyperbolic solutions, as

$$\begin{array}{c} S_{2}\left(x,t\right)=\frac{6\lambda \left(h-\rho \right){\text{sinh}}^{2}(\sqrt{-\lambda }\eta )}{j{\left(\frac{\mu }{\lambda }+\text{cosh}(\sqrt{-\lambda }\eta )\right)}^{2}}-\frac{2\lambda (h-\rho )}{j}. \end{array}$$ (33)

Again taking *B*_2_ = 0 and *B*_1_ = 1, Eq (32) produces the following hyperbolic solutions, as

$$\begin{array}{c} S_{2}\left(x,t\right)=\frac{6\lambda \left(h-\rho \right){\text{cosh}}^{2}(\sqrt{-\lambda }\eta )}{j{\left(\frac{\mu }{\lambda }+\text{sinh}(\sqrt{-\lambda }\eta )\right)}^{2}}-\frac{2\lambda (h-\rho )}{j}. \end{array}$$ (34)

The hyperbolic solutions extracted by 3^*rd*^ **solution set** are obtained, as

$$\begin{array}{c} \begin{array}{cc} S_{3}\left(x,t\right)= & -\frac{3\left(h-\rho \right)(B_{2}\sqrt{-\lambda }\text{sinh}(\sqrt{-\lambda }\eta )+B_{1}\sqrt{-\lambda }\text{cosh}(\sqrt{-\lambda }\eta ){)}^{2}}{j(B_{1}\text{sinh}(\sqrt{-\lambda }\eta )+B_{2}\text{cosh}(\sqrt{-\lambda }\eta )+\frac{\mu }{\lambda }{)}^{2}}-\frac{2\lambda (h-\rho )}{j}\\ & -\frac{3\sqrt{B_{1}^{2}-B_{2}^{2}}\sqrt{-\lambda }\left(h-\rho \right)(B_{2}\sqrt{-\lambda }\text{sinh}(\sqrt{-\lambda }\eta )+B_{1}\sqrt{-\lambda }\text{cosh}(\sqrt{-\lambda }\eta ))}{j(B_{1}\text{sinh}(\sqrt{-\lambda }\eta )+B_{2}\text{cosh}(\sqrt{-\lambda }\eta )+\frac{\mu }{\lambda }{)}^{2}}. \end{array} \end{array}$$ (35)

Setting *B*_1_ = 1 and *B*_2_ = 0, Eq (35) gives the solutions, as

$$\begin{array}{c} S_{3}\left(x,t\right)=\frac{3\lambda \left(h-\rho \right){\text{cosh}}^{2}(\sqrt{-\lambda }\eta )}{j{\left(\frac{\mu }{\lambda }+\text{sinh}(\sqrt{-\lambda }\eta )\right)}^{2}}+\frac{3\lambda \left(h-\rho \right)\text{cosh}(\sqrt{-\lambda }\eta )}{j{\left(\frac{\mu }{\lambda }+\text{sinh}(\sqrt{-\lambda }\eta )\right)}^{2}}-\frac{2\lambda (h-\rho )}{j}. \end{array}$$ (36)

Taking *B*_2_ = 1 and *B*_1_ = 0, Eq (35) gives the solutions, as

$$\begin{array}{c} S_{3}\left(x,t\right)=\frac{3\lambda \left(h-\rho \right){\text{sinh}}^{2}(\sqrt{-\lambda }\eta )}{j{\left(\frac{\mu }{\lambda }+\text{cosh}(\sqrt{-\lambda }\eta )\right)}^{2}}+\frac{3i\lambda \left(h-\rho \right)\text{sinh}(\sqrt{-\lambda }\eta )}{j{\left(\frac{\mu }{\lambda }+\text{cosh}(\sqrt{-\lambda }\eta )\right)}^{2}}-\frac{2\lambda (h-\rho )}{j}. \end{array}$$ (37)

#### 4.1.2 Case 2: λ > 0

Trigonometric solutions will be obtained in this case. The following solution sets are extracted by applying Method 1.

1^*st*^ **solution set**

$$\begin{array}{ccc} & & c_{0}=-\frac{2\lambda (h-\rho )}{j},\;c_{1}=0,\;d_{1}=\frac{3\mu (h-\rho )}{j},\;d_{2}=-\frac{3\left(h-\rho \right)\sqrt{\lambda^{2}\sigma_{2}-\mu^{2}}}{j\sqrt{\lambda }},\\ & & c_{2}=-\frac{3(h-\rho )}{j}. \end{array}$$

2^*nd*^ **solution set**

$$\begin{array}{ccc} & & c_{0}=-\frac{2\lambda (h-\rho )}{j},\;c_{1}=0,\;d_{1}=0,\;d_{2}=\frac{3\sqrt{\lambda }\sqrt{\sigma_{2}}\left(h-\rho \right)}{j},\;c_{2}=-\frac{3(h-\rho )}{j},\;\mu =0. \end{array}$$

3^*rd*^ **solution set**

$$\begin{array}{ccc} & & c_{0}=-\frac{6\lambda (h-\rho )}{j},\;c_{1}=0,\;d_{1}=0,\;d_{2}=0,\;c_{2}=-\frac{6(h-\rho )}{j},\;\mu =0. \end{array}$$

The trigonometric solutions extracted by 1^*st*^ **solution set** are obtained, as

$$\begin{array}{c} \begin{array}{cc} S_{4}\left(x,t\right)= & -\frac{3\left(h-\rho \right)\sqrt{(B_{1}^{2}+B_{2}^{2})\lambda^{2}-\mu^{2}}(B_{1}\sqrt{\lambda }\;\text{cos}\;(\sqrt{\lambda }\eta )-B_{2}\sqrt{\lambda }\;\text{sin}\;(\sqrt{\lambda }\eta ))}{j\sqrt{\lambda }(B_{1}\;\text{sin}\;(\sqrt{\lambda }\eta )+B_{2}\;\text{cos}\;(\sqrt{\lambda }\eta )+\frac{\mu }{\lambda }{)}^{2}}\\ & -\frac{3\left(h-\rho \right)(B_{1}\sqrt{\lambda }\;\text{cos}\;(\sqrt{\lambda }\eta )-B_{2}\sqrt{\lambda }\;\text{sin}\;(\sqrt{\lambda }\eta ){)}^{2}}{j(B_{1}\;\text{sin}\;(\sqrt{\lambda }\eta )+B_{2}\;\text{cos}\;(\sqrt{\lambda }\eta )+\frac{\mu }{\lambda }{)}^{2}}-\frac{2\lambda (h-\rho )}{j}\\ & +\frac{3\mu (h-\rho )}{j(B_{1}\;\text{sin}\;(\sqrt{\lambda }\eta )+B_{2}\;\text{cos}\;(\sqrt{\lambda }\eta )+\frac{\mu }{\lambda })}. \end{array} \end{array}$$ (38)

Taking *B*_1_ = 1 and *B*_2_ = 0, Eq (38) produces the following periodic solutions, as

$$\begin{array}{ccc} S_{4}\left(x,t\right) & = & -\frac{3\left(h-\rho \right)\sqrt{\lambda^{2}-\mu^{2}}\;\text{cos}\;(\sqrt{\lambda }\eta )}{j{\left(\frac{\mu }{\lambda }+\;\text{sin}\;(\sqrt{\lambda }\eta )\right)}^{2}}+\frac{3\mu (h-\rho )}{j(\frac{\mu }{\lambda }+\;\text{sin}\;(\sqrt{\lambda }\eta ))}\\ & & -\frac{3\lambda \left(h-\rho \right)\;{\text{cos}}^{2}\;(\sqrt{\lambda }\eta )}{j{\left(\frac{\mu }{\lambda }+\;\text{sin}\;(\sqrt{\lambda }\eta )\right)}^{2}}-\frac{2\lambda (h-\rho )}{j}. \end{array}$$ (39)

Again taking *B*_2_ = 1 and *B*_1_ = 0, Eq (38) produces the following periodic solutions, as

$$\begin{array}{ccc} S_{4}\left(x,t\right) & = & \frac{3\left(h-\rho \right)\sqrt{\lambda^{2}-\mu^{2}}\;\text{sin}\;(\sqrt{\lambda }\eta )}{j{\left(\frac{\mu }{\lambda }+\;\text{cos}\;(\sqrt{\lambda }\eta )\right)}^{2}}+\frac{3\mu (h-\rho )}{j(\frac{\mu }{\lambda }+\;\text{cos}\;(\sqrt{\lambda }\eta ))}\\ & & -\frac{3\lambda \left(h-\rho \right)\;{\text{sin}}^{2}\;(\sqrt{\lambda }\eta )}{j{\left(\frac{\mu }{\lambda }+\;\text{cos}\;(\sqrt{\lambda }\eta )\right)}^{2}}-\frac{2\lambda (h-\rho )}{j}. \end{array}$$ (40)

The trigonometric solutions extracted by 2^*nd*^ **solution set** are obtained, as

$$\begin{array}{c} \begin{array}{cc} S_{5}\left(x,t\right)= & -\frac{2\lambda (h-\rho )}{j}-\frac{3\left(h-\rho \right)(B_{1}\sqrt{\lambda }\;\text{cos}\;(\sqrt{\lambda }\eta )-B_{2}\sqrt{\lambda }\;\text{sin}\;(\sqrt{\lambda }\eta ){)}^{2}}{j(B_{1}\;\text{sin}\;(\sqrt{\lambda }\eta )+B_{2}\;\text{cos}\;(\sqrt{\lambda }\eta )+\frac{\mu }{\lambda }{)}^{2}}\\ & +\frac{3\sqrt{B_{1}^{2}+B_{2}^{2}}\sqrt{\lambda }\left(h-\rho \right)(B_{1}\sqrt{\lambda }\;\text{cos}\;(\sqrt{\lambda }\eta )-B_{2}\sqrt{\lambda }\;\text{sin}\;(\sqrt{\lambda }\eta ))}{j(B_{1}\;\text{sin}\;(\sqrt{\lambda }\eta )+B_{2}\;\text{cos}\;(\sqrt{\lambda }\eta )+\frac{\mu }{\lambda }{)}^{2}}. \end{array} \end{array}$$ (41)

Setting *B*_1_ = 1 and *B*_2_ = 0, Eq (41) yields the following solutions, as

$$\begin{array}{c} S_{5}\left(x,t\right)=-\frac{3\lambda \left(h-\rho \right)\;{\text{cos}}^{2}\;(\sqrt{\lambda }\eta )}{j{\left(\frac{\mu }{\lambda }+\;\text{sin}\;(\sqrt{\lambda }\eta )\right)}^{2}}+\frac{3\lambda \left(h-\rho \right)\;\text{cos}\;(\sqrt{\lambda }\eta )}{j{\left(\frac{\mu }{\lambda }+\;\text{sin}\;(\sqrt{\lambda }\eta )\right)}^{2}}-\frac{2\lambda (h-\rho )}{j}. \end{array}$$ (42)

Taking *B*_2_ = 1 and *B*_1_ = 0, Eq (41) gives the following solutions, as

$$\begin{array}{c} S_{5}\left(x,t\right)=-\frac{3\lambda \left(h-\rho \right)\;{\text{sin}}^{2}\;(\sqrt{\lambda }\eta )}{j{\left(\frac{\mu }{\lambda }+\;\text{cos}\;(\sqrt{\lambda }\eta )\right)}^{2}}-\frac{3\lambda \left(h-\rho \right)\;\text{sin}\;(\sqrt{\lambda }\eta )}{j{\left(\frac{\mu }{\lambda }+\;\text{cos}\;(\sqrt{\lambda }\eta )\right)}^{2}}-\frac{2\lambda (h-\rho )}{j} \end{array}$$ (43)

The trigonometric solutions extracted by 3^*rd*^ **solution set** are obtained, as

$$\begin{array}{c} S_{6}\left(x,t\right)=-\frac{6\left(h-\rho \right)(B_{1}\sqrt{\lambda }\;\text{cos}\;(\sqrt{\lambda }\eta )-B_{2}\sqrt{\lambda }\;\text{sin}\;(\sqrt{\lambda }\eta ){)}^{2}}{j(B_{1}\;\text{sin}\;(\sqrt{\lambda }\eta )+B_{2}\;\text{cos}\;(\sqrt{\lambda }\eta )+\frac{\mu }{\lambda }{)}^{2}}-\frac{6\lambda (h-\rho )}{j}. \end{array}$$ (44)

Inserting *B*_1_ = 1 and *B*_2_ = 0, Eq (44) yields the following periodic solutions, as

$$\begin{array}{c} S_{6}\left(x,t\right)=-\frac{6\lambda \left(h-\rho \right)\;{\text{cos}}^{2}\;(\sqrt{\lambda }\eta )}{j{\left(\frac{\mu }{\lambda }+\;\text{sin}\;(\sqrt{\lambda }\eta )\right)}^{2}}-\frac{6\lambda (h-\rho )}{j}. \end{array}$$ (45)

Again by inserting *B*_2_ = 1 and *B*_1_ = 0, Eq (44) yields the following periodic solutions, as

$$\begin{array}{c} S_{6}\left(x,t\right)=-\frac{6\lambda \left(h-\rho \right)\;{\text{sin}}^{2}\;(\sqrt{\lambda }\eta )}{j{\left(\frac{\mu }{\lambda }+\;\text{cos}\;(\sqrt{\lambda }\eta )\right)}^{2}}-\frac{6\lambda (h-\rho )}{j}. \end{array}$$ (46)

**Remark 1:** The case when λ = 0 can be performed in a similar pattern. For the sake of simplicity this case is omitted in this paper.

### 4.2 Application of method 2 for PBE

This subsection is devoted for the application of generalized projective Riccati equations method on PBE. Homogenous balancing principle gives *M* = 2. Substituting *M* = 2 in Eq (15), the following form of solution is obtained.

$$\begin{array}{c} p\left(\eta \right)=a_{0}+a_{1}A+a_{2}A^{2}+b_{1}B+b_{2}AB. \end{array}$$ (47)

Putting Eq.(4.23) into Eq.(4.3) and utilizing Method 2, the following solution sets for two cases of *ϵ* have been retrieved.

#### 4.2.1 When *ϵ* = −1

Ô Solution sets for this case are as follows:

1^*st*^ **set**

$$\begin{array}{ccc} & & a_{1}=\frac{3\delta (h-\rho )}{j},\;a_{0}=0,\;a_{2}=-\frac{3\left(h-\rho \right)(\delta^{2}+i)}{Hj},\;b_{1}=0,\;b_{2}=\frac{3\left(\rho -h\right)\sqrt{\delta^{2}+i}}{\sqrt{H}j}. \end{array}$$

2^*nd*^ **set**

$$\begin{array}{ccc} & & a_{1}=\frac{3\delta (h-\rho )}{j},\;a_{0}=\frac{H(\rho -h)}{j},\;a_{2}=-\frac{3\left(h-\rho \right)(\delta^{2}+i)}{Hj},\;b_{1}=0,\\ & & b_{2}=\frac{3\left(\rho -h\right)\sqrt{\delta^{2}+i}}{\sqrt{H}j}. \end{array}$$

The extracted hyperbolic solutions corresponding to 1^*st*^ **set** are determined as follows:

For *i* = −1,

$$\begin{array}{c} \begin{array}{cc} S_{7}\left(x,t\right)= & \frac{3\delta H\left(h-\rho \right)\;\text{sech}\;(\sqrt{H}\eta )}{j(\delta \;\text{sech}\;(\sqrt{H}\eta )+1)}-\frac{3(\delta^{2}-1)H\left(h-\rho \right)\;{\text{sech}}^{2}\;(\sqrt{H}\eta )}{j{\left(\delta \;\text{sech}\;(\sqrt{H}\eta )+1\right)}^{2}}\\ & +\frac{3\sqrt{\delta^{2}-1}H\left(\rho -h\right)\;\text{tanh}\;(\sqrt{H}\eta )\;\text{sech}\;(\sqrt{H}\eta )}{j{\left(\delta )\;\text{sech}\;(\sqrt{H}\eta )+1\right)}^{2}}. \end{array} \end{array}$$ (48)

For *i* = 1,

$$\begin{array}{c} \begin{array}{cc} S_{8}\left(x,t\right)= & \frac{3\delta H\left(h-\rho \right)\;\text{csch}\;(\sqrt{H}\eta )}{j(\delta \;\text{csch}\;(\sqrt{H}\eta )+1)}-\frac{3(\delta^{2}+1)H\left(h-\rho \right)\;{\text{csch}}^{2}\;(\sqrt{H}\eta )}{j{\left(\delta \;\text{csch}\;(\sqrt{H}\eta )+1\right)}^{2}}\\ & \frac{3\sqrt{\delta^{2}+1}H\left(\rho -h\right)\;\text{coth}\;(\sqrt{H}\eta )\;\text{csch}\;(\sqrt{H}\eta )}{j{\left(\delta \;\text{csch}\;(\sqrt{H}\eta )+1\right)}^{2}}. \end{array} \end{array}$$ (49)

The extracted hyperbolic solutions corresponding to 2^*nd*^ **set** are given as follows:

For *i* = −1,

$$\begin{array}{c} \begin{array}{cc} S_{9}\left(x,t\right)= & \frac{H(\rho -h)}{j}+\frac{3\delta H\left(h-\rho \right)\;\text{sech}\;(\sqrt{H}\eta )}{j(\delta \;\text{sech}\;(\sqrt{H}\eta )+1)}-\frac{3(\delta^{2}-1)H\left(h-\rho \right)\;{\text{sech}}^{2}\;(\sqrt{H}\eta )}{j{\left(\delta \;\text{sech}\;(\sqrt{H}\eta )+1\right)}^{2}}\\ & +\frac{3\sqrt{\delta^{2}-1}H\left(\rho -h\right)\;\text{tanh}\;(\sqrt{H}\eta )\;\text{sech}\;(\sqrt{H}\eta )}{j{\left(\delta \;\text{sech}\;(\sqrt{H}\eta )+1\right)}^{2}}. \end{array} \end{array}$$ (50)

For *i* = 1,

$$\begin{array}{c} \begin{array}{cc} S_{10}\left(x,t\right)= & \frac{H(\rho -h)}{j}+\frac{3\delta H\left(h-\rho \right)\;\text{csch}\;(\sqrt{H}\eta )}{j(\delta \;\text{csch}\;(\sqrt{H}\eta )+1)}-\frac{3(\delta^{2}+1)H\left(h-\rho \right)\;{\text{csch}}^{2}\;(\sqrt{H}\eta )}{j{\left(\delta \;\text{csch}\;(\sqrt{H}\eta )+1\right)}^{2}}\\ & +\frac{3\sqrt{\delta^{2}+1}H\left(\rho -h\right)\;\text{coth}\;(\sqrt{H}\eta )\;\text{csch}\;(\sqrt{H}\eta )}{j{\left(\delta \;\text{csch}\;(\sqrt{H}\eta )+1\right)}^{2}}. \end{array} \end{array}$$ (51)

#### 4.2.2 When *ϵ* = 1

The following solution sets are obtained for this case.

1^*st*^ **set**

$$\begin{array}{ccc} & & a_{1}=\frac{3\delta (\rho -h)}{j},\;a_{0}=0,\;a_{2}=\frac{3(\delta^{2}+i)\left(h-\rho \right)}{Hj},\;b_{1}=0,\;b_{2}=-\frac{3\iota \sqrt{\delta^{2}+i}\left(h-\rho \right)}{\sqrt{H}j}. \end{array}$$

2^*nd*^ **set**

$$\begin{array}{ccc} & & a_{1}=\frac{3\delta (\rho -h)}{j},\;a_{0}=\frac{H(h-\rho )}{j},\;a_{2}=\frac{3\left(h-\rho \right)(\delta^{2}+i)}{Hj},\;b_{1}=0,\\ & & b_{2}=-\frac{3\iota \left(h-\rho \right)\sqrt{\delta^{2}+i}}{\sqrt{H}j}. \end{array}$$

The extracted trigonometric solutions corresponding to 1^*st*^ **set** are given as follows:

For *i* = −1,

$$\begin{array}{c} \begin{array}{cc} S_{11}\left(x,t\right)= & \frac{3\delta H\left(\rho -h\right)\;\text{sec}\;(\sqrt{H}\eta )}{j(\delta \;\text{sec}\;(\sqrt{H}\eta )+1)}+\frac{3(\delta^{2}-1)H\left(h-\rho \right)\;{\text{sec}}^{2}\;(\sqrt{H}\eta )}{j{\left(\delta \;\text{sec}\;(\sqrt{H}\eta )+1\right)}^{2}}\\ & \frac{3\iota \sqrt{\delta^{2}-1}H\left(h-\rho \right)\;\text{tan}\;(\sqrt{H}\eta )\;\text{sec}\;(\sqrt{H}\eta )}{j{\left(\delta \;\text{sec}\;(\sqrt{H}\eta )+1\right)}^{2}}. \end{array} \end{array}$$ (52)

The extracted trigonometric solutions corresponding to 2^*nd*^ **set** are given as follows:

For *i* = −1,

$$\begin{array}{c} \begin{array}{cc} S_{12}\left(x,t\right)= & \frac{H(h-\rho )}{j}+\frac{3\delta H\left(\rho -h\right)\text{sec}(\sqrt{H}\eta )}{j(\delta \text{sec}(\sqrt{H}\eta )+1)}+\frac{3(\delta^{2}-1)H\left(h-\rho \right){\text{sec}}^{2}(\sqrt{H}\eta )}{j{\left(\delta \text{sec}(\sqrt{H}\eta )+1\right)}^{2}}\\ & -\frac{3\iota \sqrt{\delta^{2}-1}H\left(h-\rho \right)\text{tan}(\sqrt{H}\eta )\text{sec}(\sqrt{H}\eta )}{j{\left(\delta \text{sec}(\sqrt{H}\eta )+1\right)}^{2}}. \end{array} \end{array}$$ (53)

**Remark 2:** It is important to mention here that the similar nature of trigonometric solutions can be obtained by taking *ϵ* = 1 and *i* = 1. Moreover, by taking *ϵ* = 0, the rational solutions can be obtained. For the sake of simplicity, these cases have been omitted in this paper.

## 5 Graphical illustration

**Fig 1:**
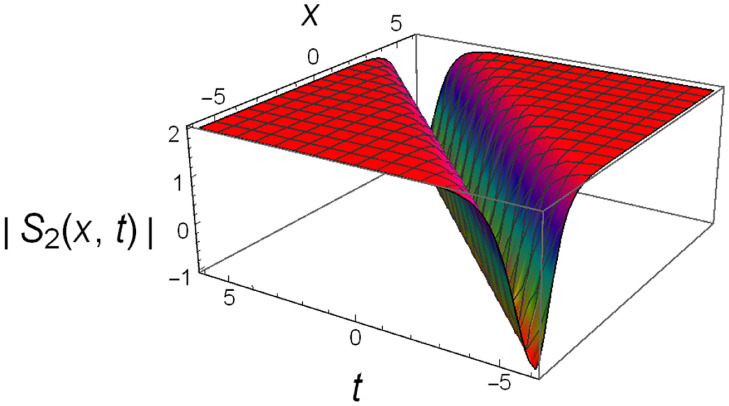
Surface plot of ∣*S*_2_(*x*, *t*)∣ for λ = −1, *ρ* = −2.3, *j* = 2.7, *h* = 1.78, *v* = 4, *μ* = 0, *B*_1_ = 0, *B*_2_ = 1.

**Fig 2:**
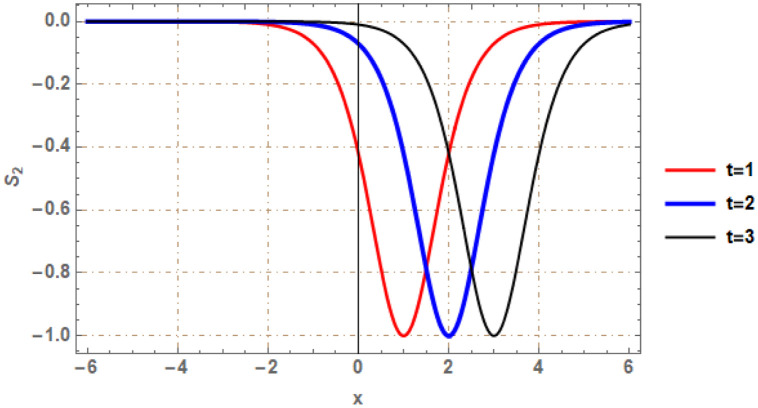
2D line plot of ∣*S*_2_(*x*, *t*)∣ for λ = −1, *ρ* = −2.3, *j* = 2.7, *h* = 1.78, *v* = 4, *μ* = 0, *B*_1_ = 0, *B*_2_ = 1.

**Fig 3:**
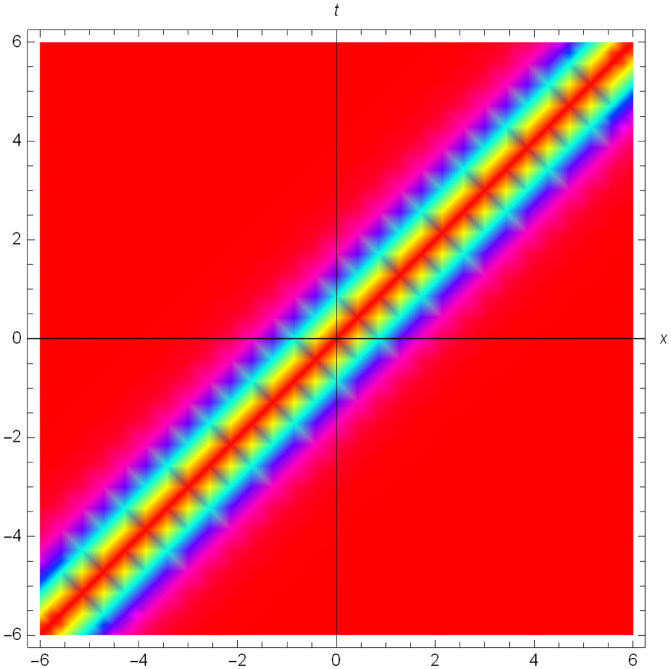
Density plot of ∣*S*_2_(*x*, *t*)∣ for λ = −1, *ρ* = −2.3, *j* = 2.7, *h* = 1.78, *v* = 4, *μ* = 0, *B*_1_ = 0, *B*_2_ = 1.

**Fig 4:**
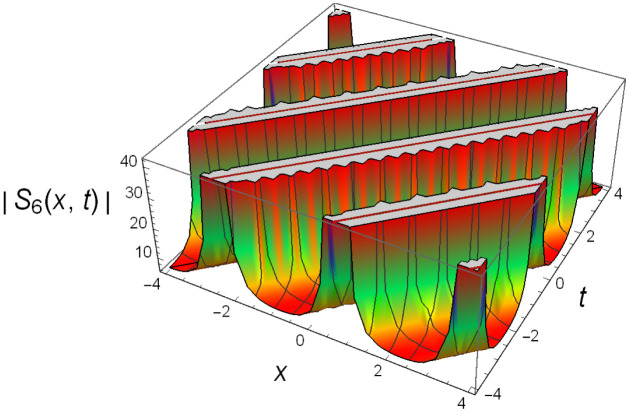
Surface plot of ∣*S*_6_(*x*, *t*)∣ for λ = 1.5, *ρ* = −0.45, *j* = −2.7, *h* = −3.1, *v* = 4, *μ* = 0, *B*_1_ = 0, *B*_2_ = 1.

**Fig 5:**
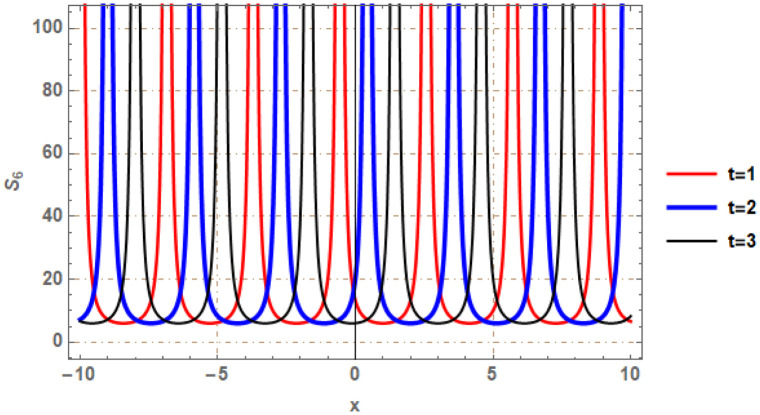
2D line plot of ∣*S*_6_(*x*, *t*)∣ for λ = 1.5, *ρ* = −0.45, *j* = −2.7, *h* = −3.1, *v* = 4, *μ* = 0, *B*_1_ = 0, *B*_2_ = 1.

**Fig 6:**
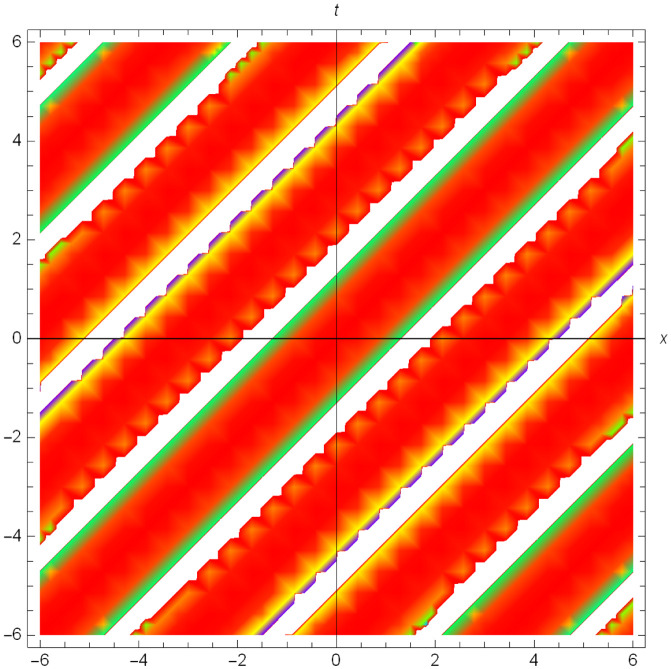
Density plot of ∣*S*_6_(*x*, *t*)∣ for λ = 1.5, *ρ* = −0.45, *j* = −2.7, *h* = −3.1, *v* = 4, *μ* = 0, *B*_1_ = 0, *B*_2_ = 1.

**Fig 7:**
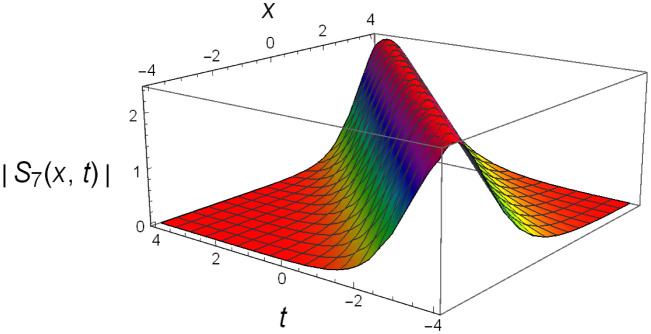
Graphs of ∣*S*_7_(*x*, *t*)∣ for *δ* = 3.5, *ρ* = −4.3, *j* = 3.1, *h* = −2.5, *v* = 2, *H* = 3.3.

**Fig 8:**
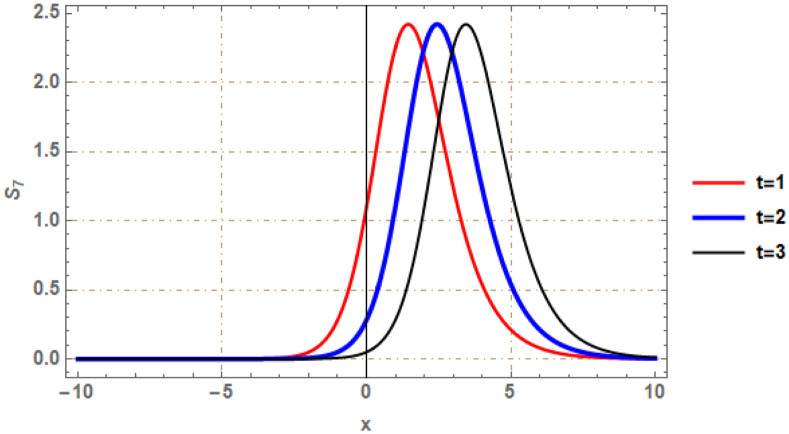
2D line plot of ∣*S*_7_(*x*, *t*)∣ for *δ* = 3.5, *ρ* = −4.3, *j* = 3.1, *h* = −2.5, *v* = 2, *H* = 3.3.

**Fig 9:**
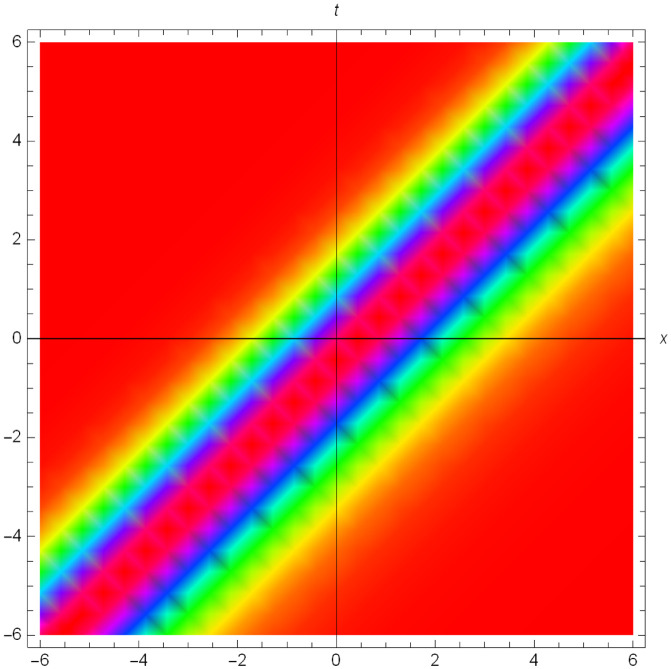
Density plot of ∣*S*_7_(*x*, *t*)∣ for *δ* = 3.5, *ρ* = −4.3, *j* = 3.1, *h* = −2.5, *v* = 2, *H* = 3.3.

**Fig 10:**
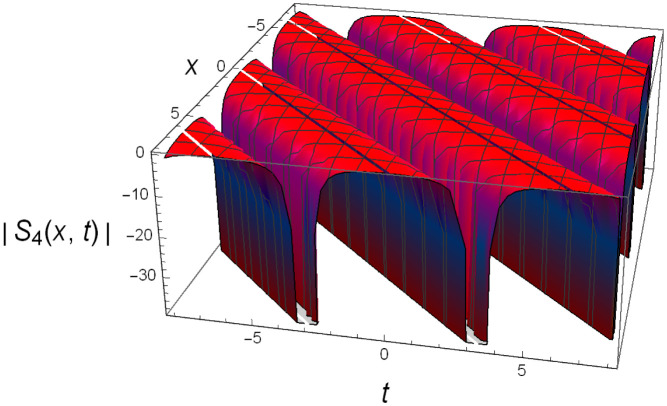
Graphs of ∣*S*_4_(*x*, *t*)∣ for *μ* = 0, *ρ* = 1, *j* = 2, *h* = 2, *v* = 1, λ = 1.

**Fig 11:**
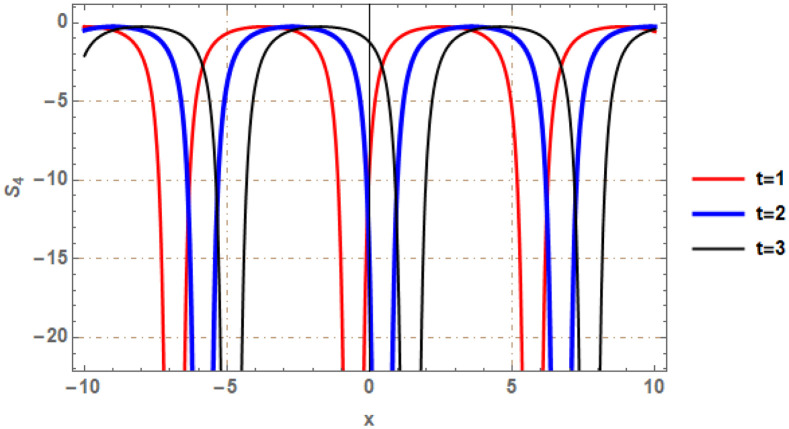
2D line plot of ∣*S*_4_(*x*, *t*)∣ for *μ* = 0, *ρ* = 1, *j* = 2, *h* = 2, *v* = 1, λ = 1.

**Fig 12:**
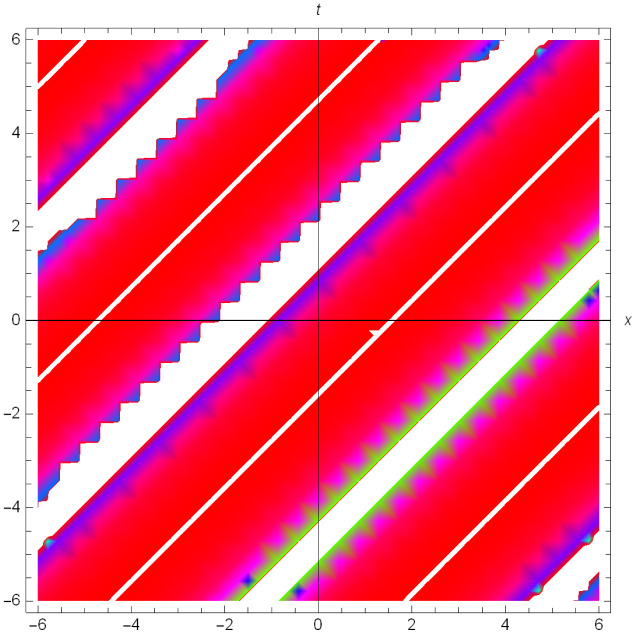
Density plot of ∣*S*_4_(*x*, *t*)∣ for *μ* = 0, *ρ* = 1, *j* = 2, *h* = 2, *v* = 1, λ = 1.

Graphical illustrations of few of the extracted solutions are provided in this section. The solutions that are obtained above indicate wave structures of different forms. These different wave structures can be proven useful in nonlinear evolution models. Therefore, to understand these varying wave structures, graphical illustrations are considered to be very effective. The exact solutions are presented by the aid of 2D, 3D, and density plots. The surface, line and density plots of *S*_2_(*x*, *t*) are displayed in Figs 1–3, respectively. The surface, line and density plots of *S*_6_(*x*, *t*) are displayed in Figs 4–6, respectively. The surface, line and density plots of *S*_7_(*x*, *t*) are displayed in Figs 7–9, respectively. The surface, line and density plots of *S*_4_(*x*, *t*) are displayed in Figs 10–12, respectively.

## 6 Conclusion

The perturbed Boussinesq equation has been studied in this article using two distinct methods: the two variables $\left(\frac{G^{\prime }}{G},\frac{1}{G}\right)$ expansion method and the generalized projective Riccati equations method. These methodologies have demonstrated their efficacy not only in the context of the perturbed Boussinesq equation but also across various other nonlinear partial differential equations. Few limitations and restrictions are associated with every analytical technique. Techniques applied in this research too obey some limitations. By following the given restrictions on the proposed techniques we are successfully extract bright solitons, dark soliton, periodic solutions, singular solitons and combo solitons solution for the governing model. The applied approaches are very efficient in obtaining new soliton solutions for variety of NLPDEs. These methods retrieved diverse form of solutions, containing trigonometric, rational and hyperbolic expressions. On comparing our finding with [23–25], it has been found that the results obtained in this paper using suggested methods are new and have not been reported earlier in literature. To enhance the understanding of the physical properties of these solutions we have explained the existence of these bright solitons, singular solitons, bright singular solitons, and periodic soliton solutions, through graphical representations employing appropriately chosen arbitrary parameters. Moreover, density plots have been meticulously provided to facilitate a deeper comprehension of the obtained solutions. It is noteworthy that the results presented in this study are novel, offering fresh insights into the dynamics of shallow water, coastal, and oceanic waves. They stand as robust tools that can significantly augment the study and understanding of these complex phenomena.

## References

[1] Yang Y., Qi J. M., Tang X. M., and Gu Y. Y. Further results about traveling wave exact solutions of the (2 + 1)-dimensional modified KdV equation. Advances in Mathematical Physics, 2019:3053275, 2019.

[2] Akbar M. A., Ali N. H. M., and Tanjim T. Adequate soliton solutions to the perturbed boussinesq equation and the KdV-Caudrey-Dodd-Gibbon equation. Journal of King Saud University Science, 32(6):2777–2785, 2020. doi: 10.1016/j.jksus.2020.06.014

[3] Sulaiman T. A., Bulut H., Yokus A., and Baskonus H. M. On the exact and numerical solutions to the coupled Boussinesq equation arising in ocean engineering. Indian journal of Physics, 93(5):647–656, 2019. doi: 10.1007/s12648-018-1322-1

[4] Akram G., Sadaf M., and Dawood M. Abundant soliton solutions for Radhakrishnan-Kundu-Laksmanan equation with Kerr law non-linearity by improved *ϕ*(*ξ*)/2-expansion technique. International Journal for Light and Electron Optics, 247:167787, 2021. doi: 10.1016/j.ijleo.2021.167787

[5] Arshed S., Biswas A., Alzahrani A.K., and Belic M. Solitons in nonlinear directional couplers with optical metamaterials by first integral method. Optik-International Journal for Light and Electron Optics, 218:165208, 2020. doi: 10.1016/j.ijleo.2020.165208

[6] Akram G., Sadaf M., and Zainab I. New graphical observations for Kdv equation and Kdv-Burgers equation using modified auxiliary equation method. Modern Physics Letters B, 36:2150520, 2021. doi: 10.1142/S0217984921505205

[7] Kumar Sachin, Niwas Monika, Osman M S, and Abdou M A. Abundant different types of exact soliton solution to the (4+1)-dimensional Fokas and (2+1)-dimensional breaking soliton equations. Communications in Theoretical Physics, 73(10):1–17, 2021. doi: 10.1088/1572-9494/ac11ee

[8] Niwas M. and Kumar S. Multi-peakons, lumps, and other solitons solutions for the (2+1)- dimensional generalized benjamin-ono equation: an inverse-$\frac{G^{\prime }}{G}$-expansion method and real-world applications. Nonlinear Dynamics, 111(24):22499–22512, 2023. doi: 10.1007/s11071-023-09023-3

[9] Kumar S. and Niwas M. Analyzing multi-peak and lump solutions of the variable-coefficient Boiti-Leon-Manna-Pempinelli equation: a comparative study of the lie classical method and unified method with applications. Nonlinear Dynamics, 111(24):22457–22475, 2023. doi: 10.1007/s11071-023-09012-6

[10] Kumar S., Hamid I., and Abdou M.A. Dynamic frameworks of optical soliton solutions and soliton-like formations to Schrödinger-Hirota equation with parabolic law non-linearity using a highly efficient approach. Opt Quant Electron, 55:1261, 2023. doi: 10.1007/s11082-023-05461-w

[11] Rani S., Kumar S., and Mann N. On the dynamics of optical soliton solutions, modulation stability, and various wave structures of a (2+1)-dimensional complex modified Korteweg-de-Vries equation using two integration mathematical methods. Opt Quant Electron, 55:731, 2023. doi: 10.1007/s11082-023-04946-y

[12] Hamid I. and Kumar S. Symbolic computation and novel solitons, traveling waves and soliton like solutions for the highly nonlinear (2+1)-dimensional Schrödinger equation in the anomalous dispersion regime via newly proposed modified approach. Opt Quant Electron, 55:755, 2023. doi: 10.1007/s11082-023-04903-9

[13] Asjad M. I., Hamood Rehman H. U., Ishfaq Z., Awrejcewicz J., Akgü A., and Riaz M. B. On soliton solutions of perturbed Boussinesq and KdV-Caudery-Dodd-Gibbon equations. Coatings, 11(1429):1429, 2021. doi: 10.3390/coatings11111429

[14] Ebadi Q., Johnson S., Zerrad E., and Biswas A. Solitons and other nonlinear waves for the perturbed Boussinesq equation with power law nonlinearity. Journal of King Saud University Science, 24(3):237–241, 2012. doi: 10.1016/j.jksus.2011.05.001

[15] Jiao X. Y. Truncated series solutions to the (2+1)-dimensional perturbed Boussinesq equation by using the approximate symmetry method. Chinese Physics B, 27(10):100202, 2018. doi: 10.1088/1674-1056/27/10/100202

[16] Duran S. Solitary wave solutions of the coupled Konno-Oono equation by using the functional variable method and the two variables (G’/G, 1/G)-expansion method. Adiyaman University Journal of Science, 10(2):585–594, 2020.

[17] Al-Shawba A. A., Abdullah F. A., Gepreel K. A., and Azmi A. Solitary and periodic wave solutions of higher-dimensional conformable time-fractional differential equations using the $\left(\frac{G^{\prime }}{G},\frac{1}{G}\right)$ expansion method. Advances in Difference Equations, 2018(1):1–15, 2018. doi: 10.1186/s13662-018-1814-5

[18] Akram G., Arshed S., Sadaf M., and Sameen F. The generalized projective Riccati equations method for solving quadratic-cubic conformable time-fractional Klien-Fock-Gordon equation. Ain Shams Engineering Journal, 13(2022):101658, 2021.

[19] Akram G., Sadaf M., Arshed S., and Sameen F. Bright, dark, kink, singular and periodic soliton solutions of Lakshmanan-Porsezian-Daniel model by generalized projective Riccati equations method. Optik: International Journal for Light and Electron Optics, 241:167051, 2021. doi: 10.1016/j.ijleo.2021.167051

[20] Almatrafi M., A. A., and Cemil Tunç. Constructions of the soliton solutions to the good boussinesq equation. Advances in Difference Equations, 2020, 11 2020.

[21] Zhijian Yang. On local existence of solutions of initial boundary value problems for the “bad” boussinesq-type equation. Nonlinear Analysis: Theory, Methods and Applications, 51(7):1259–1271, 2002. doi: 10.1016/S0362-546X(01)00894-X

[22] Li M., Zhang W., and Wu Q. Analytical and numerical results on global dynamics of the generalized Boussinesq equation with cubic nonlinearity and external excitation. Mathematical Problems in Engineering, 2021:6629095, 2021.

[23] Demiray Seyma Tuluce and Bayrakci Ugur. Novel solutions of perturbed boussinesq equation article. Journal of Mathematical Sciences and Modelling, 5:99–104, 11 2022. doi: 10.33187/jmsm.1123178

[24] Nisar Kottakkaran Sooppy, Akinyemi Lanre, Inc Mustafa, Şenol Mehmet, Mirzazadeh Mohammad, Houwe Alphonse, Abbagari Souleymanou, et al. New perturbed conformable boussinesq-like equation: Soliton and other solutions. Results in Physics, 33:105200, 2022. doi: 10.1016/j.rinp.2022.105200

[25] Jiao Xiao-Yu. Truncated series solutions to the (2+1)-dimensional perturbed boussinesq equation by using the approximate symmetry method. Chinese Physics B, 27(10):100202, oct 2018. doi: 10.1088/1674-1056/27/10/100202

